# Protein lactylation in metabolic dysfunction-associated steatotic liver disease: a mechanistic review

**DOI:** 10.1186/s13098-026-02105-3

**Published:** 2026-02-09

**Authors:** Huilin Chen, Yuntao Ye, Tianmu He, Bo Li, Qiang Wang

**Affiliations:** 1 Clinical Medical College, Southwest Medical University, 646000 Luzhou, Sichuan China; 2 Department of Hepatobiliary Surgery, Affiliated Hospital of Southwest Medical University, 646000 Luzhou, Sichuan China; 3 Department of Nephrology, First Medical Center of Chinese PLA General Hospital, Haidian District, 100853 Beijing, China

**Keywords:** Protein lactylation, Metabolic dysfunction-associated steatotic liver disease, Lactate, Metabolism, Epigenetics

## Abstract

Metabolic dysfunction-associated steatotic liver disease (MASLD) is a prevalent chronic liver disorder with limited therapeutic options. While its pathogenesis is complex, the underlying molecular drivers remain incompletely understood. Recently, protein lactylation, a novel lactate-derived post-translational modification (PTM), has emerged as a key regulator linking metabolic reprogramming to cellular function. This review elucidates the pivotal roles of protein lactylation throughout the progression of MASLD, from simple steatosis to metabolic dysfunction-associated steatohepatitis (MASH) and hepatocellular carcinoma, establishing a conceptual framework centered on the “lactate-lactylation-gene expression” axis and examining its dysregulation across hepatocytes, hepatic stellate cells, and immune cells throughout MASLD. Furthermore, we discuss emerging therapeutic strategies targeting the axis, aiming to provide a theoretical foundation for the development of precision medicine for MASLD.

## Introduction

### MASLD: a global health challenge with limited therapeutic options

Metabolic dysfunction-associated steatotic liver disease (MASLD), previously known as non-alcoholic fatty liver disease (NAFLD), is an acquired metabolic stress liver injury related to insulin resistance and genetic susceptibility, including simple fatty liver (SFL), metabolic dysfunction-associated steatohepatitis (MASH) and related cirrhosis [1]. Substantial fat deposition in hepatocytes causes SFL in the early stage of MASLD, then with increasing lipid accumulation, lipotoxicity and mitochondrial dysfunction is prompted, triggering hepatocyte death, inflammation and fibrosis, leading to MASH, finally cirrhosis and hepatocellular carcinoma (HCC) [2]. The overall prevalence of MASLD worldwide was about 32.4% [3] and that’s projected to continue rising [4]. This trend discloses that MASLD does increasing harm to human health. Therefore, exploring novel pathogenic mechanisms and identifying new therapeutic targets are of vital importance in treating MASLD.

The etiology of MASLD is multifactorial, involving a complex interplay of genetic, environmental, and metabolic factors. Recent researches have revealed that the MASLD development is associated with specific genetic variants, among which the most prominent are patatin like domain 3, 1-acylglycerol-3-phosphate O-acyltransferase (PNPLA3) and transmembrane 6 superfamily member 2 (TM6SF2) [5]. Individuals carrying variants in 17-beta hydroxysteroid dehydrogenase 13 (HSD17B13) and glucokinase regulator (GCKR) also have an increased genetic susceptibility to MASLD according to the genome-wide association studies (GWAS) [6]. Additionally, environmental factors, including sedentary lifestyles and high-calorie diets as well as consumption of high-fat diets and sugar-sweetened beverages, exacerbate the risk of MASLD [7]. Besides, environmental toxins, like pesticides and industrial chemicals, have been implicated in propelling the process of MASLD as well [8]. Emerging evidence also demonstrates the role of gut microbiota dysbiosis in driving hepatic inflammation and insulin resistance, probably further contributing to disease pathogenesis [9].

The pathogenesis of MASLD embraces a cascade of molecular and cellular events. Under above influences, there is hepatic lipid accumulation subsequently, resulting from an imbalance between lipid influx and apolipoprotein transport/hepatocyte oxidative decomposition. Concurrent insulin resistance (IR) exacerbates steatosis, which is frequently associated with hyperlactatemia, both as a consequence of altered systemic and hepatic glucose metabolism, providing a substrate pool for protein lactylation [10]. Lipid overload induces endoplasmic reticulum and oxidative stress, leading to hepatocyte injury and apoptosis [11]. This damage, along with factors like mitochondrial dysfunction, pro-fibrogenic factors, and Kupffer cell activation, drives chronic inflammation [10]. Ultimately, activated hepatic stellate cells (HSCs) promote fibrosis, progressing towards cirrhosis [12]. Interestingly, mechanisms like carcinoembryonic antigen-related cell adhesion molecule 1 (CEACAM1) loss can drive fibrosis independently of IR and steatosis [13]. Furthermore, aberrant epigenetic reprogramming and epitranscriptomic remodeling also affect MASLD progression [14].

Despite advances in understanding MASLD pathogenesis, effective therapeutic options remain limited. Lifestyle modifications, such as weight loss and dietary changes, are the cornerstone of management, but adherence is often poor [15]. Bariatric surgery, including the methods of vertical gastrectomy and Roux-en-Y gastric bypass (RYGB), has become an effective way to treat certain cases of obesity, which is possibly able to reduce the risk of MASLD [16]. Pharmacological interventions, including vitamin E, pioglitazone and resmetirom, have shown modest efficacy in specific patients, but their long-term safety and effectiveness are debated [17, 18]. Therefore, it is urgent to research novel therapeutic targets and strategies by comprehensively understanding the process of MASLD and revealing the key mechanisms.

### Protein lactylation: a novel link between metabolism and pathology

Lactate, a byproduct of glycolysis, is widely recognized as a metabolic substrate that provides energy. In 2019, Zhang et al. [19] introduced the concept of histone lysine lactylation (Kla), demonstrating that lactate plays a role in regulating cellular functions under pathophysiological conditions.

Lactylation is a post-translational modification (PTM), serving various important functions like epigenetic regulation, immune modulation, cellular metabolism, and diverse biological processes. By directly modulating histones or transcription factors, lactylation alters chromatin structure and gene expression. For example, H3K18la facilitates *Arg1* expression during M1 macrophage polarization to repair tissues [19], while lactylation of *p53* impairs its tumor-suppressive function [20]. In immune regulation, histone lactylation facilitates the transition of macrophages to a reparative phenotype to suppress inflammatory responses [21] and modulates CD8 (+) T cell function [22]. Lctylation can regulate energy metabolism in cells as well. Hypoxia-induced alanyl-tRNA synthetase 2 (AARS2) lactylates PDHA1 and carnitine palmitoyltransferase 2 (CPT2), inhibiting oxidative phosphorylation—a process reversible by SIRT3 [23]. Finally, lactylation may interact with other modifications, such as acetylation, exhibiting both competitive and synergistic relationships on shared targets like HMGB1 [24–26].

Lactylation is critically implicated in diverse pathologies, including cardiovascular diseases, cancers, and neurodegenerative disorders. Following myocardial infarction, elevated H3K18la in macrophages promotes reparative gene expression (e.g., *Lrg1*,* Vegf-a*,* IL-10*) for cardiac repair, while SNAIL1 lactylation may conversely drive fibrosis [24, 27]. In cancer, AARS1-mediated lactylation of *p53* at K120/K139 inactivates its tumor-suppressive function, a process inhibitable by β-alanine [20]; NBS1 lactylation also enhances homologous recombination repair, conferring chemoresistance [28]. In Alzheimer’s disease models, H4K12la enrichment in microglia establishes a glycolysis/H4K12la/PKM2 positive feedback loop, exacerbating microglial dysfunction and disease progression [29].

Long-term lipid accumulation disrupts in vivo metabolic homeostasis, a process often associated with elevated circulating lactate levels [30]. The precise impact of this altered metabolic signature on MASLD progression, however, remains to be fully elucidated. Notably, circulating lactate elevation is closely linked to systemic IR, a core feature of metabolic syndrome that underpins MASLD. Given that lactate is the substrate for protein lactylation, elevated lactate levels in MASLD patients suggest that lactylation may serve as a critical mechanistic link connecting metabolic dysregulation to the pathological changes observed in the disease. Therefore, in the context of MASLD, a central “lactate-lactylation-gene expression” axis emerges, where metabolic dysregulation leads to lactate accumulation, which in turn drives protein lactylation to epigenetically and functionally reprogram key liver cells, thereby fueling disease pathogenesis. It is crucial to note that the functional outcomes of this axis are highly context-dependent, with evidence pointing to both disease-promoting and homeostatic roles. This review will summarize the documented and proposed roles of the axis in MASLD and discuss emerging therapeutic strategies that target this pathway.

## The fundamentals of protein lactylation

### The lactate-lactylation axis

Lactylation is a PTM distinguished by the covalent attachment of a lactate group to lysine residues on target proteins [31]. Under hypoxic conditions or bacterial infection, both of intracellular lactate and histone Kla levels are elevated; whereas by inhibiting activities of lactate dehydrogenase (LDH) and pyruvate dehydrogenase (PDH), lactate production is diminished and histone Kla levels are lowered as well. This mechanistic relationship suggests the positive correlation between endogenous lactate concentrations and histone Kla levels [19]. This establishes a fundamental “lactate-lactylation axis”—metabolic perturbations (e.g., enhanced glycolysis, mitochondrial dysfunction) lead to lactate accumulation, which in turn drives lactylation modification on target proteins. Hitherto lactylation was initially identified on histone lysine residues, for instance, H3K18la, H4K5la, and H2BK6la [19, 32]. However, subsequent lactylome studies have revealed that lactylation is far more prevalent on non-histone proteins, encompassing a wide array of metabolic enzymes, transcription factors, and signaling molecules [33, 34]. For example, Fan et al. [27] demonstrated that lactate upregulates endothelial-mesenchymal transition (EndoMT) following myocardial infarction through lactylation of SNAIL1, thereby exacerbating cardiac fibrosis and aggravating ventricular dysfunction.

### Regulatory mechanisms: writers, erasers, and readers

As a dynamic and reversible PTM, lactylation is conceptually regulated by three classes of proteins: writers, erasers, and readers. Writers add lactyl groups to target proteins, while erasers mediate their removal; readers recognize and bind lactylated residues to modulate downstream signaling pathways, thus regulating gene expression and cellular processes. Notably, p300—a histone acetyltransferase with broad substrate specificity—has been proposed as a lactyltransferase based on cellular overexpression and knockdown experiments, though its specificity and physiological role require further validation [19]. Subsequent studies identified other putative writers, such as GCN5 [24] and HBO1 [35]. In vitro studies suggest that histone deacetylase 1–3 (HDAC1-3) and silent information regulator 1–3 (SIRT1-3) may possess delactylase activity, with HDAC1 and HDAC3 specifically shown to remove ε-N-lactoyllysine on histones in cellular models [36]. However, the in vivo relevance and substrate spectrum of these enzymes remain areas of active investigation. As for readers, Hu et al. [37] performed proteomic analysis of H3K18la immunoprecipitation experiment during induced pluripotent stem cell (iPSC) reprogramming, uncovering the specific recruitment of Brg1 to lactylated histones, establishing its role as a lactylation reader. Additionally, non-enzymatic protein lactylation has also been documented under specific conditions [38]. Despite these advances, current understanding of lactylation sites, molecular mechanisms, and associated proteins remains limited, necessitating further investigations.

### The Dichotomous/Context-Dependent role of protein lactylation in MASLD

Emerging evidence reveals that protein lactylation functions not as a simplistic binary “switch” but as a sophisticated “molecular rheostat” that finetunes cellular responses in MASLD. Its ultimate biological impact is highly context-dependent, governed by three critical determinants——target protein, cell type as well as disease stage. For instance, H3K18la in HSCs promotes a pro-fibrotic gene program, representing a central epigenetic mechanism driving liver fibrosis, which may correlate with MASLD [25]. In this context, inhibiting lactylation presents a clear therapeutic rationale. Conversely, it has been reported that lactylation of the fatty acid synthase (FASN) at K673 in hepatocytes is associatively linked to inhibition of its enzymatic activity, thereby reducing *de novo* lipogenesis (DNL) and potentially alleviating steatosis [39]. This suggests a compensatory or protective role for specific lactylation events. Therefore, future therapeutic strategies aimed at targeting lactylation must exhibit a high degree of specificity, with the precise goal of “correcting” detrimental lactylation events while preserving or even enhancing beneficial ones.

## Pathogenic roles of protein lactylation in MASLD

Emerging evidences show that proteins involved in hepatocytes activities are regulated by lactylation [40]. Therefore, dysregulated protein lactylation plays an important role in the process of MASLD.

### In hepatocytes: driving steatosis and metabolic dysfunction

**Fig. 1:**
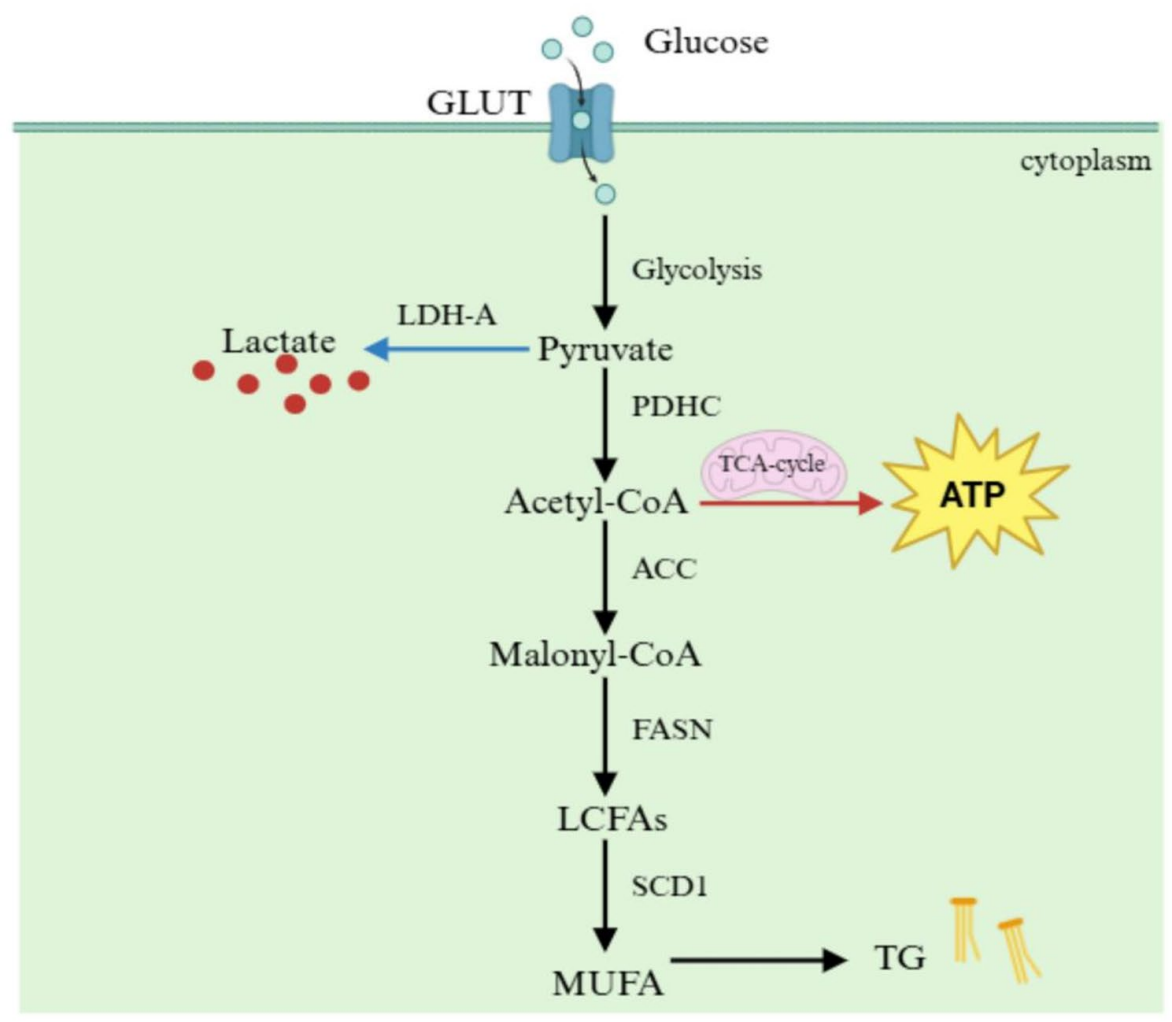
*De novo* lipogenesis: Hepatocytes utilize acetyl-CoA to synthesize fatty acids, which are subsequently esterified to form triglycerides. This figure is created in BioRender. GLUT: glucose transporter; LDH-A: lactate dehydrogenase-A; PDHC: pyruvate dehydrogenase complex; TCA: tricarboxylic acid; ATP: adenosine triphosphate; ACC: acetyl-CoA carboxylase; FASN: fatty acid synthase; LCFAs: long-chain fatty acids; SCD1: stearoyl-CoA desaturase 1; MUFA: monounsaturated fatty acid; TG: triglycerides

Disordered hepatic lipid metabolism is the primary initiating factor in the development of MASLD [10]. Among these mechanisms, DNL represents one of the key pathways driving lipid accumulation [41]. The substrate for DNL, acetyl-CoA, is mainly derived from glycolysis [42]. Pyruvate generated through glycolysis follows two main pathways: some is converted into lactate via LDH-A catalysis, while the majority is transformed into acetyl-CoA to fuel energy production through the tricarboxylic acid (TCA) cycle [43]. Under conditions of excess acetyl-CoA production or impaired glucose metabolism, acetyl-CoA is carboxylated by acetyl-CoA carboxylase (ACC) to form malonyl-CoA [44]. Subsequently, malonyl-CoA serves as a substrate for FASN, which catalyzes the synthesis of long-chain saturated fatty acids [45]. These saturated fatty acids are further modified by stearoyl-CoA desaturase 1 (SCD1) to generate monounsaturated fatty acids (MUFA) [46], which ultimately undergo esterification to form triglycerides (TG), thereby promoting lipid accumulation [47] **(**Fig. 1**)**. Consequently, molecular targets associated with DNL regulation hold potential as therapeutic strategies for MASLD [48–50].

FASN, a multifunctional homodimeric enzyme, is a key regulator of DNL [51]. Currently, FASN is recognized as a novel therapeutic target for MASLD, with several FASN inhibitors such as SNX8 and TRIM56, demonstrating potential in alleviating MASLD pathology [52–54]. The mitochondrial pyruvate carrier (MPC) is a critical carrier protein, transporting pyruvate into mitochondria for TCA cycle. Serving as a metabolic hinge, MPC is implicated in various diseases [55]. Gao et al. [39] reported elevated MPC1 levels accompanied by reduced FASN lactylation (primarily at the K673 site) in MASLD patients, correlating with hepatic lipid deposition. The proposed mechanism suggests that MPC1 upregulation enhances pyruvate entry into mitochondria, reducing subsequent lactate production, which means lactate deficiency limits lactylation substrate availability, leading to diminished FASN lactylation. Since lactylation has been correlated with suppressed FASN activity in vitro, the observed reduction in FASN K673la is hypothesized to enhance FASN enzymatic function, potentially contributing to lipid synthesis and accumulation. But a direct causal relationship between FASN K673 lactylation and enzymatic inhibition in the context of MASLD pathogenesis awaits experimental confirmation. This MPC1/FASN lactylation axis illustrates a potential feed-forward loop where metabolic shifts away from lactate production may inadvertently exacerbate steatosis by reducing inhibitory lactylation of FASN.

SCD1, an integral membrane protein anchored in the endoplasmic reticulum, catalyzes the conversion of saturated fatty acids into MUFA, primarily oleate and palmitoleate [56]. As a central lipogenic enzyme, SCD1 is recognized as a potential therapeutic target for regulating DNL [56]. Meng et al. [57] proposed that a histone lactylation/N6-methyladenosine (m6A) modification axis promotes MASLD progression through SCD1 upregulation. Mechanistically, in hepatocytes exposed to free fatty acids and in mice fed a high-fat diet, upregulated LDH-A increases lactate production, which enhances H3K18la enrichment in the proximal promoter region of methyltransferase-like 3 (METTL3). This leads to increased METTL3 expression and subsequent m6A methylation of SCD1 mRNA. The m6A modification is recognized by YTHDF1, stabilizing SCD1 transcripts, elevating SCD1 protein levels, and amplifying DNL and lipid accumulation. This pathway illustrates how lactate-derived histone lactylation can epigenetically reinforce lipogenic gene expression through epitranscriptomic regulation, thereby linking metabolic flux to transcriptional output during hepatic steatosis.

Protein lactylation can also disrupt hepatic lipid metabolism by inducing lipid peroxidation. For instance, high-alcohol-producing Klebsiella pneumoniae (HiAlc Kdn) in the intestinal microbiota increases global histone lactylation levels in hepatocytes, triggering lipid peroxidation. This process leads to mitochondrial damage, impaired cell proliferation, and increased apoptosis [58]. These effects may contribute to chronic liver injury, though further studies are required to validate this hypothesis.

### In hscs: facilitating fibrosis in MASH

Prolonged lipid metabolism dysfunction not only induces hepatic steatosis but also triggers hepatocyte inflammation and injury, driving the process from simple SFL to MASH [10]. In advanced MASH, activated HSCs further promote hepatic fibrosis [59]. HSC activation refers to their transformation from a quiescent state into myofibroblasts, characterized by high expression of α-smooth muscle actin (α-SMA) and excessive extracellular matrix (ECM) deposition [60]. To meet heightened energy demands, HSCs undergo series of metabolic adaptations, with metabolic reprogramming—primarily marked by enhanced aerobic glycolysis—being a key mechanism [60, 61]. Therefore, lactate, a byproduct of glycolysis, may play a significant role in advancing hepatic fibrosis.

Hexokinase 2 (HK2), the first enzyme in glycolysis, catalyzes the phosphorylation of hexoses and is a key enzyme for glycolysis. Numerous studies have identified HK2 as a potential therapeutic target for various diseases [62–64]. Recent research further suggests that HK2 may also represent a promising therapeutic target for hepatic fibrosis [25]. During HSC activation, upregulated HK2 enhances glycolysis and lactate production, which in turn elevates H3K18la. It preferentially enriches at the promoter regions of activation-induced genes in HSCs primarily *α-SMA* and collagen type I alpha 1 (COL1α1), promoting their expression and thereby contributing to HSC activation and hepatic fibrosis. Importantly, H3K18la competes with H3K18ac at the same lysine residue; class I HDAC inhibitors suppress HSC activation by elevating H3K18ac and displacing H3K18la. This establishes a HK2/glycolysis/H3K18la positive feedback loop that sustains the profibrotic phenotype of HSCs, illustrating how metabolic reprogramming in HSCs exploits lactylation as an epigenetic mechanism to perpetuate liver fibrosis.

Aldolase, fructose-bisphosphate A (ALDOA), is an another key enzyme in glycolysis, catalyzing the reversible conversion of β-D-fructose 1,6-bisphosphate into triose phosphates. Zhou et al. [65] reported that ALDOA is a downstream target of insulin-like growth factor 2 mRNA-binding protein 2 (IGF2BP2), with IGF2BP2 regulating ALDOA expression to mediate its biological functions in glycolytic metabolism and HSCs activation. Specifically, during hepatic fibrosis, upregulation of IGF2BP2 increases ALDOA expression, promoting glycolysis and lactate production. This subsequently induces global lactylation in hepatic tissues and H3K18la, which exacerbates fibrosis by enhancing LX-2 cell migration and α-SMA expression.

SRY-box transcription factor 9 (SOX9), is a highly conserved transcription factor which plays a critical role in regulating hepatocyte fate determination and tissue morphogenesis [66]. A recent study proposed that histone lactylation accelerates hepatic fibrosis progression by promoting SOX9 expression [67]. Silencing LDH-A in LX-2 cells reduced H3K18la levels and suppressed SOX9 expression, accompanied by downregulation of fibrosis-associated genes (*α-SMA* and *COL1α1*). However, SOX9 overexpression rescued the effect.

### In MASLD-Associated HCC (MASLD-HCC): a potential oncogenic driver

The incidence of HCC secondary to MASLD is approximately 15.1%, with a notable proportion of these cases occurring in non-cirrhotic livers [68, 69]. Mechanistically, both genetically determined MASLD and the inflammatory processes related to MASH may have potential roles in HCC development [70]. Current evidences suggest that MASLD progression to HCC involves activation of multiple signaling pathways [71]. MASLD is associated with insulin resistance, resulting in elevated levels of insulin and insulin-like growth factor-1 (IGF-1), which activate the phosphoinositide 3-kinases/protein kinase B (PI3K/AKT) and mitogen-activated protein kinase (MAPK) pathways through binding to their respective receptors [72]. This molecular cascade ultimately leads to inhibition of hepatocyte apoptosis, induction of cellular proliferation, and hepatic hyperplasia, collectively promoting hepatocarcinogenesis [73]. A recent study reported that pyrroline-5-carboxylate reductase 1 (PYCR1), a key enzyme in proline metabolism, is upregulated in HCC and correlated with poor prognosis. Knockdown or pharmacological inhibition of PYCR1 suppresses the development of HCC [74]. PYCR1 expression is significantly positively correlated with glycolysis. Therefore, when PYCR1 is downregulated, reduced lactate production from glycolysis decreases H3K18la enrichment in the insulin receptor substrate 1 (IRS1) gene promoter region. This leads to decreased IRS1 expression and subsequently lowers the levels of key proteins in the IRS1 downstream signaling pathways—PI3K/AKT/mTOR and MAPK/ERK. Consequently, activation of these pathways is inhibited, ultimately suppressing hepatocellular carcinoma cell proliferation and metastasis, and delaying HCC progression. These findings highlight histone lactylation as a context-dependent amplifier of HCC, linking glycolytic metabolism to oncogenic signaling through an epigenetic mechanism.

**Fig. 2:**
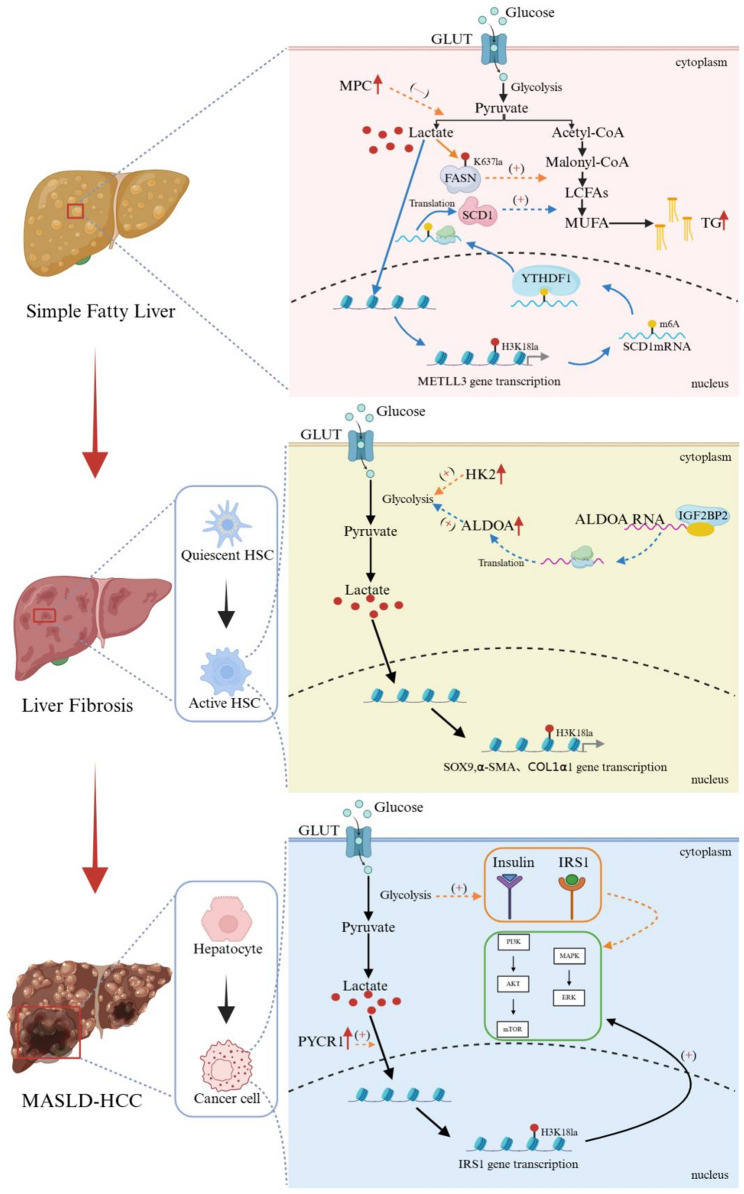
The “lactate-lactylation-gene expression” axis in MASLD pathogenesis. Schematic summarizing how lactate, derived from enhanced glycolysis, drives protein lactylation to differentially regulate gene expression and cellular functions in hepatocytes, HSCs, and cancer cells during the progression from simple fatty liver to fibrosis and hepatocellular carcinoma. This figure is created in BioRender. GLUT: glucose transporter; MPC: mitochondrial pyruvate carrier; FASN: fatty acid synthase; SCD1: stearoyl-CoA desaturase 1; LCFAs: long-chain fatty acids; MUFA: monounsaturated fatty acid; TG: triglycerides; METTL3: methyltransferase-like 3; H3K18la: histone H3 lysine 18 lactylation; HSC: hepatic stellate cell; HK2: hexokinase 2; ALDOA: aldolase, fructose-bisphosphate A; IGF2BP2: insulin-like growth factor 2 mRNA-binding protein 2; SOX9: SRY-box transcription factor 9; α-SMA: α-smooth muscle actin; COL1α1: collagen type I alpha 1; MASLD-HCC: MASLD-associated HCC; IRS1: insulin receptor substrate 1; PYCR1: pyrroline-5-carboxylate reductase 1; PI3K/AKT/mTOR: phosphoinositide 3-kinases/protein kinase B/mammalian target of rapamycin; MAPK/ERK: mitogen-activated protein kinase/extracellular signal-regulated kinase

In summary, protein lactylation emerges as a critical molecular nexus in MASLD pathogenesis. It acts not only as a downstream effector of disordered lipid metabolism via lactate accumulation but also as an upstream regulator that extensively interacts with metabolic reprogramming, immune dysregulation, and mitochondrial dysfunction, thus underscoring its pleiotropic role in driving the disease (Fig. 2 and Table 1). The mechanisms across hepatocytes, HSCs, and cancer cells illustrate a unifying theme: metabolic reprogramming like glycolysis upregulation increases lactate, which drives specific lactylation events to activate transcriptional programs that can promote steatosis, fibrosis, and carcinogenesis, respectively. Conversely, in specific contexts like FASN lactylation, it may play a protective role. Thus, lactylation is best understood as an integrative mechanism that intersects with and modulates multiple established axes of MASLD pathology.

**Table 1:** Lactylation of target proteins assosicated with MASLD

Stage of MASLD	Target protein	Lactylation site/type	Upstream regulator/driver	Downstream mechanism	Pathophysiological role in MASLD progression	Reference
**I. Simple Fatty Liver**	FASN	K673la	Reduced MPC1 activity.	Reduced lactylation enhances FASN function in DNL.	Promotes DNL and hepatic lipid accumulation.	[55]
	Histone H3 (regulating SCD1)	H3K18la	Increased lactate production driven by upregulated LDH-A.	Increases SCD1 protein expression by METTL3/m6A/YTHDF1 axis	Accelerates DNL and TG synthesis, exacerbating steatosis.	[57]
	Global Histones	Global lactylation	Gut microbiota-derived factors.	Triggers lipid peroxidation, resulting in mitochondrial damage, impaired cell proliferation, and increased apoptosis.	Contributes to chronic liver injury and inflammation.	[58]
**II. MASH ** **/ Fibrosis**	Histone H3 (in HSCs)	H3K18la	Enhanced glycolysis in HSCs driven by HK2.	Enriches at the promoters of profibrotic genes to activate their transcription.	Drives HSC activation and ECM deposition, promoting fibrosis.	[35]
	Histone H3 (in HSCs)	H3K18la, Global lactylation	Upregulated ALDOA expression, mediated by IGF2BP2.	Promotes the expression of fibrosis markers and enhances HSC migration.	Exacerbates hepatic fibrosis.	[65]
	Histone H3 (regulating *SOX9* in HSCs)	H3K18la	LDH-A-mediated lactate production in HSCs.	Activates the transcription of *SOX9*, subsequently upregulateing *α-SMA* and *COL1α1*.	Accelerates hepatic fibrosis progression.	[67]
**III. MASLD-HCC**	Histone H3 (regulating *IRS1* in cancer cells)	H3K18la	PYCR1-driven enhancement of glycolysis.	Increased H3K18la enhances *IRS1* transcription, leading to hyperactivation of downstream PI3K/AKT/mTOR and MAPK/ERK signaling pathways.	Promotes proliferation and metastasis of hepatocellular carcinoma cells.	[74]

## Therapeutic strategies targeting protein lactylation

### Modulation via natural compounds

Emerging research on protein lactylation in MASLD has identified multiple natural compounds that ameliorate MASLD by modulating histone lactylation. Given the critical role of Kla in macrophage M1 polarization and the chronic liver injury characteristic of MASLD, salvianolic acid B (Sal B) has been reported to inhibit macrophage M1 polarization by reducing LDH-A expression and lowering H3K18la levels at the LDH-A gene promoter, potentially mitigating liver injury [75]. Yin’s team [32] demonstrated that Jianpi Huazhuo Tiaozhi Granules, a herbal formulation, effectively reduce hepatic lipid accumulation by elevating H2BK6la and H4K80la lactylation levels in hepatocytes, offering a promising therapeutic strategy for MASLD. Additionally, irigenin, a monomeric anthocyanin, improves MASH by mediating tRF-31R9J. This tRNA-derived fragment recruits HDAC1 to reduce histone lactylation and acetylation modifications at promoters of pro-ferroptosis genes (*ATF3*, *ATF4*, *CHAC1*), thereby suppressing their expression and inhibiting hepatocyte ferroptosis, ultimately alleviating MASH [76]. Nevertheless, it is notable that above researches are only based on animal experiments and lack related clinical reports, which can serve as one of the prospective research directions.

### Targeting specific lactylation sites and pathways

The therapeutic targeting of lactylation presents a unique challenge due to its context-dependent roles. As discussed, inhibiting lactylation (e.g., H3K18la in HSCs) may be beneficial for fibrosis, while promoting specific lactylation events (e.g., FASN K673la) might alleviate steatosis. Therefore, future therapeutic strategies must be highly precise, aiming to selectively correct pathogenic lactylation while preserving or augmenting protective modifications. On one hand, targeting histone lactylation may ameliorate MASLD progression [57, 67]. For instance, Rho et al. [25] demonstrated through in vitro and in vivo studies that HSC2-specific or systemic HK2 gene deletion alleviates hepatic fibrosis by reducing H3K18la levels without hepatotoxicity. On the other hand, non-histone lactylation modifications, such as FASN K673la, are also linked to MASLD progression. Researchers have proposed the MPC1/FASN lactylation axis as a potential target for MASLD intervention [39]. Specifically, knockdown of MPC1 may elevate lactate levels and enhance FASN lactylation, which in turn reduces FASN enzymatic activity, thereby alleviating lipid accumulation.

### Regulating upstream metabolic pathways

MASLD can also be ameliorated by modulating cellular energy metabolism. For example, there is indirect evidence that SIRT3 may mediate protein delactylation. Moreover, other studies also propose that SIRT3 can reverse AARS2-mediated lactylation of PDH and CPT2, thereby maintaining cellular oxidative phosphorylation and ensuring normal energy supply. Modulating SIRT3 activity thus presents a strategy to counteract the metabolic suppression caused by excessive lactylation under hypoxic or glycolytic conditions. These mechanisms provide novel therapeutic strategies for improving hepatocyte metabolic dysfunction in MASLD [23].

To sum up, based on the mechanism by which protein lactylation drives the progression of MASLD, therapeutic interventions that simultaneously mitigate lipid accumulation and target protein lactylation could constitute an ideal strategy for improving clinical outcomes in MASLD, but needing further research **(**Table 2**)**.

**Table 2:** Innovative therapeutic strategies targeting lactylation in MASLD

Therapeutic strategy/agent	Primary target(s)	Mechanism of action	Pathophysiological outcome in MASLD	Evidence level	Reference
**Natural Compounds**					
Salvianolic acid B	H3K18la	Inhibits LDH-A expression, thereby reducing H3K18la levels at the LDH-A gene promoter and suppressing macrophage M1 polarization.	Mitigation of chronic liver injury.	Preclinical	[75]
Jianpi Huazhuo Tiaozhi Granules	H2BK6la; H4K80la	Elevates the levels of H2BK6la and H4K80la in hepatocytes.	Reduction of hepatic lipid accumulation.	Preclinical	[22]
Irigenin	tRNA-derived fragment tRF-31R9J; HDAC1	Mediates tRF-31R9J to recruit HDAC1, which reduces histone lactylation at the promoters of pro-ferroptosis genes (*ATF3*,* ATF4*,* CHAC1*).	Alleviation of MASH by inhibiting hepatocyte ferroptosis.	Preclinical	[76]
**Genetic/Molecular Targeting**					
Genetic deletion of HK2	H3K18la	Reduces lactate production by inhibiting glycolysis, leading to decreased H3K18la levels.	Alleviation of hepatic fibrosis.	In vitro & in vivo	[35]
Targeting the MPC1/FASN axis (e.g., MPC1 knockdown)	MPC1; FASN lactylation (K673la)	MPC1 knockdown increases cytosolic lactate, enhancing FASN lactylation and subsequently reducing its enzymatic activity.	Alleviation of hepatic lipid accumulation.	Preclinical	[55]
**Metabolic Modulation**					
Modulation of SIRT3 activity	Lactylated PDH and CPT2	Acts as a protein delactylase, reversing AARS2-mediated lactylation of PDH and CPT2 to maintain normal oxidative phosphorylation.	Improvement of hepatocyte metabolic dysfunction.	In vitro	[34]

## Challenges and future directions

Despite rapid progress, the field of protein lactylation in MASLD faces several key challenges that must be addressed to translate mechanistic insights into clinical practice. Many studies, including those on FASN K673la [39] and the HK2/H3K18la axis in fibrosis [25], report strong associations. Future work employing cell-specific knockout models, lactylation-site-specific mutants, and pharmacological modulation in vivo is crucial to establish causality. Besides, most evidence derives from animal models or cell lines, lacking human validation. Studies quantifying the lactylome in human liver biopsies across the MASLD spectrum are urgently required to confirm pathophysiological relevance and identify human-specific biomarkers. Additionally, incomplete characterization of enzymatic machinery is another challenge. The identities of physiological “writers” (e.g., definitive validation of p300) and “erasers” (e.g., confirming SIRT3’s role in vivo) remain partially resolved. The discovery and validation of specific “readers” beyond Brg1 are also needed. Last but not least, since both increasing and decreasing lactylation can be beneficial, depending on the target and disease stage, this creates a significant challenge for drug development, demanding extreme specificity to avoid off-target effects that could counteract the intended benefit.

Building on the identified challenges, future research should focus on following aspects. Firstly, large-scale proteomic studies of lactylation in well-characterized human MASLD cohorts are essential to map disease-stage-specific modifications and correlate them with clinical parameters. Dynamic changes in specific lactylation marks hold promise as non-invasive biomarkers for MASLD staging and fibrosis risk assessment. Furthermore, genetic and chemical biological approaches are needed to definitively identify and characterize the major lactyltransferases and delactylases operating in hepatocytes, HSCs, and liver immune cells, which includes exploring the potential of developing selective delactylase modulators. At last, a growing body of research has integrated clinical data with artificial intelligence (AI) techniques, particularly machine learning and deep learning, to explore the diagnosis and staging of MASLD and other hepatic disorders, uncover their underlying biological mechanisms, and identify novel biomarkers [77–79]. Future studies, by integrating multi-omics data including genomics, transcriptomics, proteomics, metabolomics, as well as clinical information, could construct AI models to identify key pathways and molecular drivers that contribute to the progression of MASLD, such as the development of selective lactyltransferase or delactylase modulators and the integration of multi-omics data with artificial intelligence to decipher the lactylation code in MASLD. Overcoming these hurdles will be crucial for establishing lactylation-targeted approaches as a cornerstone of precision medicine for MASLD and related metabolic disorders.

## Conclusion

MASLD represents a significant global health burden characterized by lipid accumulation, inflammation, and fibrosis, with limited therapeutic options. Mechanistically, disordered lipid metabolism elevates lactate levels and enhances histone and non-histone lactylation, collectively regulating MASLD progression. In addition, lactylation modifications interact with metabolic reprogramming, immune dysregulation, and mitochondrial dysfunction, underscoring their multifaceted regulatory role in MASLD pathogenesis. In summary, a dual-pronged approach that etiologically addresses lipid deposition while simultaneously targeting protein lactylation may represent a promising therapeutic strategy to improve MASLD outcomes. Nevertheless, the efficacy and safety of this combined approach require extensive experimental validation.

## Data Availability

No data was used for the research described in the article.

## Abbreviations

MASLD: Metabolic dysfunction-associated steatotic liver disease
NAFLD: Non-alcoholic fatty liver disease
SFL: Simple fatty liver
MASH: Dysfunction-associated steatohepatitis
HCC: Hepatocellular carcinoma
PNPLA3: Patatin like domain 3, 1-acylglycerol-3-phosphate O-acyltransferase
TM6SF2: Transmembrane 6 superfamily member 2
HSD17B13: 17-beta hydroxysteroid dehydrogenase 13
GCKR: Glucokinase regulator
GWAS: Genome-wide association studies
IR: Insulin resistance
HSCs: Hepatic stellate cells
CEACAM1: Cell adhesion molecule 1
RYGB: Roux-en-Y gastric bypass
Kla: lysine lactylation
PTM: Post-translational modification
AARS2: Alanyl-tRNA synthetase 2
CPT2: Carnitine palmitoyltransferase 2
HMBG1: High mobility group box-1 protein
LDH: Lactate dehydrogenase
PDH: Pyruvate dehydrogenase
EndoMT: Endothelial-mesenchymal transition
HDAC1-3: Histone deacetylase 1-3
SIRT1-3: Silent information regulator 1-3
IPSC: Induced pluripotent stem cell
FASN: Fatty acid synthase
DNL: De novo lipogenesis
TCA: Tricarboxylic acid
ACC: Acetyl-CoA carboxylase
SCD1: Stearoyl-CoA desaturase 1
MUFA: Monounsaturated fatty acids
TG: Triglycerides
MPC: Mitochondrial pyruvate carrier
m6A: N6-methyladenosine
METTL3: Methyltransferase-like 3
HiAlc Kdn: High-alcohol-producing Klebsiella pneumoniae
α-SMA: α-smooth muscle actin
ECM: Excessive extracellular matrix
HK2: Hexokinase 2
COL1α1: Collagen type I alpha 1
ALDOA: Aldolase, fructose-bisphosphate A
IGF2BP2: Insulin-like growth factor 2 mRNA-binding protein 2
SOX9: SRY-box transcription factor 9
MASLD-HCC: MASLD-associated HCC
IGF-1: insulin-like growth factor-1
PI3K/AKT: Phosphoinositide 3-kinases/protein kinase B
MAPK: Mitogen-activated protein kinase
PYCR1: Pyrroline-5-carboxylate reductase 1
IRS1: Pnsulin receptor substrate 1
Sal B: Salvianolic acid B
CPT2: Carnitine palmitoyltransferase 2
AI: Artificial intelligence
